# Comprehensive analysis of histone deacetylases genes in the prognosis and immune infiltration of glioma patients

**DOI:** 10.18632/aging.204071

**Published:** 2022-05-11

**Authors:** Lin Shen, Yanyan Li, Na Li, Liangfang Shen, Zhanzhan Li

**Affiliations:** 1 Department of Oncology, Xiangya Hospital, Central South University, Changsha 410008, Hunan Province, PR China; 2 Department of Nursing, Xiangya Hospital, Central South University, Changsha 410008, Hunan Province, PR China; 3 National Clinical Research Center for Geriatric Disorders, Xiangya Hospital, Central South University, Changsha 410008, Hunan Province, PR China

**Keywords:** glioma, histone deacetylases, bioinformatics, prognostic factor, immune infiltration

## Abstract

The occurrence and development of tumors are closely related to histone deacetylases (HDACs). However, their relationship with the overall biology and prognosis of glioma is still unknown. In the present study, we developed and validated a prognostic model for glioma based on HDAC genes. Glioma patients can be divided into two subclasses based on eleven HDAC genes, and patients from the two subclasses had markedly different survival outcomes. Then, using six HDAC genes (HDAC1, HDAC3, HDAC4, HDAC5, HDAC7, and HDAC9), we established a prognostic model for glioma patients, and this prognostic model was validated in an independent cohort. Furthermore, the calculated risk score from six HDACA genes expression was found to be an independent prognostic factor that could predict the five-year overall survival of glioma patients well. High-risk patients have changes in multiple complex functions and molecular signaling pathways, and the gene alterations of high- and low-risk patients were significantly different. We also found that the different survival outcomes of high- and low-risk patients could be related to the differences in immune filtration levels and the tumor microenvironment. Subsequently, we identified several small molecular compounds that could be favorable for glioma patient treatment. Finally, the expression levels of HDAC genes from the prognostic model were validated in glioma and nontumor tissue samples. Our results revealed the clinical utility and potential molecular mechanisms of HDAC genes in glioma. A model based on six HDAC genes can predict the overall survival of glioma patients well, and these genes are potential therapeutic targets.

## INTRODUCTION

Glioma is deemed to be the most aggressive tumor in the central nervous system. The annual incidence of glioma has been reported to be approximately 30–80/1 million worldwide and increasing by 1–2% annually; the 5-year survival rate is only 10–20% [1]. According to the WHO standard, glioma is divided into four grades by its pathological characteristics [2]: grade I, pilocytic astrocytoma, which manifests as a benign tumor, and patients may have full clinical recovery after total tumor resection. Grade II has a poor prognosis compared with grade I but is still considered to be a low-grade glioma. Grade III, such as anaplastic astrocytoma, as well as grade IV and GBM, are types of advanced grade glioma with a high degree of malignancy, strong invasive ability, a poor prognosis and multiple differentiation potentials, and the median survival time is only approximately one year [3].

Although various cancer therapies have been applied over the past decades, the prognosis of glioma patients remains dismal. After including the isocitrate dehydrogenase (IDH1/2) mutation and whether the 1p19q code is missing, the WHO classification of central nervous system tumors has become more refined [4]. However, the clinical outcomes and side effects of patients with the same grade and classification of tumors are not the same after being comprehensively treated [5]. This suggests that we still need to explore more instructive molecular targets of glioma to reveal its unclear and complex molecular mechanisms.

The underlying cause of malignant tumors is disordered gene expression systems, including oncogenes, tumor suppressor genes and genes related to DNA repair [6]. With the continuous development of epigenetics, it has been gradually recognized that almost all malignant tumors also have epigenetic abnormalities, which, together with gene changes, cause tumorigenesis [7]. The epigenetic phenomena involved in the occurrence of malignant tumors mainly include abnormal DNA methylation, histone modification and their interaction caused by abnormal expression of noncoding RNA and chromosomal remodeling [8]. These epigenetic changes lead to abnormal activation of certain genes and silencing, thereby allowing cell growth to enter an uncontrolled state.

The occurrence and development of tumors are closely related to histone deacetylases (HDACs) [9]. Studies have shown that genome-wide histone acetylation levels are generally reduced in tumor cells, among which HDAC1, HDAC5 and HDAC7 are regarded as tumor markers [10]. Second, studies have shown that gene knockout of HDAC1/2 in breast cancer cells or HDAC1/2/3 in colon cancer cells can induce tumor cell apoptosis, suggesting that the activity of HDACs is related to tumor cell survival [11, 12]. Similarly, abnormal binding of HDACs to oncogene fusion proteins at certain gene loci is also regarded as an important mechanism of tumorigenesis.

HDACs come in four classes: class I (HDAC1, HDAC2, and HDAC3, HDAC8), class II (HDAC4, HDAC5, HDAC6, HDAC7, HDAC9, and HDAC10), class III (SIRT1-SIRT7), and class IV (HDAC11) [13]. A previous study explored the roles of HDACs in the prognosis of clear cell renal cell carcinoma [14]. However, their roles in the overall biology and prognosis of glioma are still unknown. In the present study, we comprehensively explored the biological function and prognosis of eleven HDAC genes in glioma, which may contribute to a better understanding of the underlying molecular mechanisms and identify potential therapeutic targets for glioma patients.

## MATERIALS AND METHODS

### Data source

The mRNA expression data of glioma patients from The Cancer Genome Atlas (TCGA, https://portal.gdc.cancer.gov/) and Chinese Glioma Genome Atlas (CGGA, http://www.cgga.org.cn/) were utilized. Gene mutation data were also obtained from the TCGA database. Drug response data were acquired from the Genomics of Drug Sensitivity in Cancer (GDSC) database (https://www.cancerrxgene.org/downloads). The immune filtration data were from TCIA (https://tcia.at/home). The information on the histone deacetylase genes (HDAC1, HDAC2, HDAC3, HDAC4, HDAC5, HDAC6, HDAC7, HDAC8, HDAC9, HDAC10, and HDAC11) was from the Molecular Signatures Database (MSigDB).

### Clustering analysis

Using the R “Consensus Cluster Plus” package, we performed clustering analysis. Consensus Cluster Plus allows data clustering with a negative value. Using the K-means method, we achieved the most approximate number of clusters by extracting 1000 times from 80% of the sample size. The results are presented using a consensus matrix heatmap. We also used principal component analysis (PCA) and t-distributed stochastic neighbor embedding (tSNE) to further validate the clustering analysis.

### Development and validation of the HDAC gene prognostic model

Using 11 HDAC genes, we developed an overall survival (OS) prediction model in the TCGA dataset. Least absolute shrinkage and selection operator (LASSO) regression was used to select the number entering the model, and then a multivariate Cox regression was performed to obtain the regression coefficient of each HDAC gene. We calculated the risk score of each sample using the following formula: risk score = coef_1_ × gene_1_ expression + coef_2_ × gene_2_ expression + coef_n_ × gene_n_ expression. Using the established prognostic model, we performed validation in the CGGA dataset. Glioma patients were separated into high- and low-risk groups according to the median risk score. Kaplan–Meier survival curves were used to compare the difference in OS between the high- and low-risk groups. Receiver operating characteristic curves (ROCs) were used to evaluate the predictive ability at 1 year, 2 years, and 3 years of patient OS in TCGA and CGGA. PCA was used to identify the risk type.

### Clinical correlation and independent analysis

To investigate the association between the risk score and prognosis in glioma patients, we first performed a stratified analysis of different clinical parameters. Then, we compared the difference in HDAC genes between the two clustering groups, and the risk score distributions were also observed for different clinical parameters. To validate the independence of the risk score, we performed univariate and multivariate Cox regression analyses by adjusting the clinical parameters (TCGA: age, sex, grade; CGGA: age, sex, grade, history, TNM stage, radiotherapy, chemotherapy, occurrence type, IDH and 1p19q status). We evaluated the diagnostic ability of the risk score and other parameters using ROC curves. We built a nomogram to evaluate the clinical application of the HDAC gene prognostic model, and the nomogram-predicted probabilities of 1-year OS, 3-year OS and 5-year OS were used to assess the model fitting ability.

### Functional, pathway enrichment and mutation analyses

To explore the function and pathway enrichment of different high- and low-risk groups, we performed GO functional enrichment and KEGG pathway analysis using the “clusterProfiler” package. Using the masked copy number segmentation data, we investigated the gene mutation frequency of different risk groups using the “maftool” package (gene alteration, variant classification, variant type, co-occurrence and mutually exclusive).

### Immune filtration, tumor microenvironment, and drug sensitivity analysis

We assessed the difference in 16 immune cell-related infiltrating scores and 13 immune-related pathways between the high- and low-risk groups. Using the TCGA dataset, we calculated the immune and stromal estimate scores of the high- and low-risk groups using the R “estimate” package. To explore the correlation between small molecular drugs and the identified prognostic signature genes, Pearson correlation coefficients were calculated. |R|>0.25 and *P* < 0.05 were considered significantly correlated.

### Validation of HDAC genes in glioma and nontumor tissue

To validate the expression of six HDAC genes, including the prognostic model (HDAC1, HDAC3, HDAC4, HDAC5, HDAC7, and HDAC9), we detected the expression of these HDAC genes in glioma and nontumor tissues. mRNA expression data were collected from 23 samples from epilepsy patients and 157 tumor samples (GEO dataset). The tissue collection was approved by the NCI IRB committee, and informed consent was obtained from all subjects [15].

### Statistical analysis

Differentially expressed gene analysis was performed using the “limma” package. Differences for category variables were performed using the chi-square test. Comparisons of OS curves were achieved using the log-rank test. One-way ANOVA was used to compare the differences in HDAC gene expression among nontumor and different grades of glioma. SNK methods were used for multiple comparisons. All statistical analyses were performed using R software 4.0.1, and *P* < 0.05 was considered significant.

### Data availability

The TCGA data can be obtained from the https://portal.gdc.cancer.gov/, and CGGA data can be available from the Chinese Glioma Genome Atlas (http://www.cgga.org.cn/). Some data have been provided in the Supplementary Table 1. The expression levels of HDAC genes from non-tumor and glioma patients can be available from the GEO (GSE4290).

### Ethics approval

The ethnic approval is granted because these data were from public database.

## RESULTS

### Identification of two subclasses in glioma

**Figure 1:**
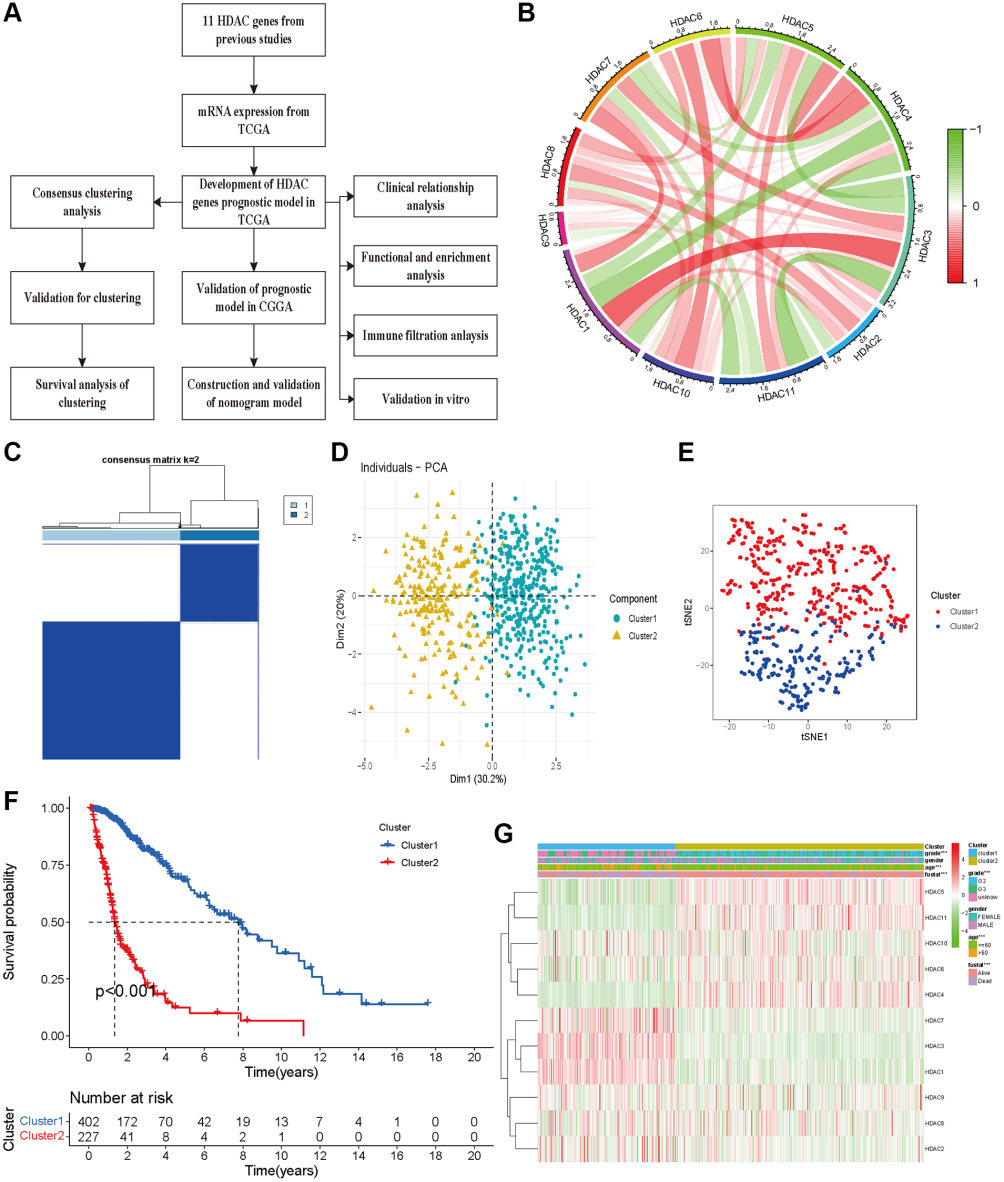
**Glioma patients can be separated into two subclasses using HDAC genes.** (**A**) The flow chart of data analysis. (**B**) The correlation circle plot among eleven HDAC genes. (**C**) The consensus matrix plot identified the best grouping (k = 2). (**D**) Principal component analysis of glioma subclasses in the TCGA dataset. (**E**) The corrected t-SNE2 analysis for two subclasses. (**F**) The Kaplan-Meier survival curve for two subclasses in TCGA dataset. (**G**) The correlation of different clinical parameters and HDAC gens expressions with subclasses.

A flow chart was constructed to comprehensively describe our study (Figure 1A). For 11 HDAC genes, the correlations among the HDAC members were different. HDAC6 and HDAC8 showed a positive association with other HDAC genes, while HDAC3 and HDAC4 showed a negative correlation with other HDAC genes (Figure 1B). We performed clustering analysis using 11 HDAC genes (HDAC1-HDAC11). The consistency coefficient was calculated to achieve the optimal clustering number (K value), and k = 2 was finally selected as the optimal clustering number. The sharp and clear boundaries showed stable and robust clustering for glioma patients (Figure 1C). To validate the two subclasses, we further performed individual PCA and t-SNE with decreased dimensions of features. We found that the glioma patients were well distributed into two components (Figure 1D and Supplementary Table 2, Cluster 1 and Cluster 2), and t-SNE also suggested that the samples presented a two-dimensional distribution model (Figure 1E). The Kaplan–Meier survival curve indicated that Cluster 2 had worse OS than Cluster 1 (Figure 1F). The clustering group was also associated with some clinical parameters (age, sex, grade and survival outcomes). HDAC1, HDAC4, HDAC5, HDAC6 and HDAC10 were highly expressed in Cluster 2, and HDAC1, HDAC2, HDAC3, HDAC7, and HDAC9 were significantly highly expressed in Cluster 1 (Figure 1G).

### Development and validation of an HDAC gene prognostic model for glioma

**Figure 2:**
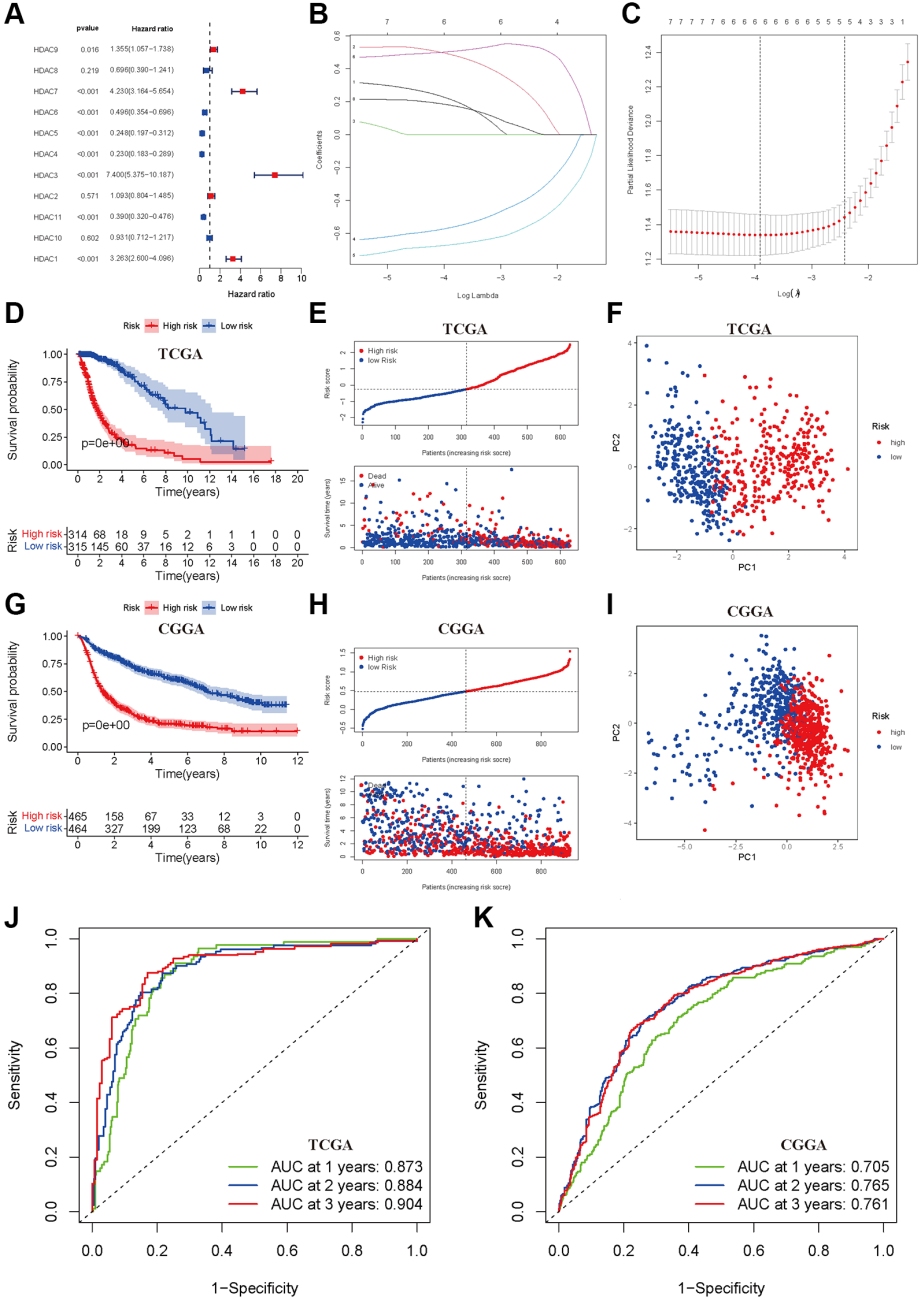
**Development and validation of prognostic model based on HDAC genes.** (**A**) Forest plot of univariate cox regression for HDAC genes in glioma patients. (**B**) LASSO regression of the 11 OS-related HDAC genes. (**C**) Cross-validation for turning parameters selection in the LASSO regression. (**D**) Kaplan-Meier survival curve of high- and low-risk groups from developed prognostic model based on 6 HDAC genes in TCGA. (**E**) Distributions of risk scores and survival time of glioma patients in TCGA. (**F**) PCA plot for high- and low-risk group in TCGA. (**G**) Kaplan-Meier survival curve of high- and low-risk groups from validated prognostic model based on 6 HDAC genes in CGGA. (**H**) Distributions of risk scores and survival time of glioma patients in CGGA. (**I**) PCA plot for high- and low-risk group in CGGA. (**J** and **K**) The receiver operating characteristic curve for predicting 1-year, 2-year, and 3-year survival rate of glioma patients in TCGA and CGGA.

We first developed a prognostic model of OS in the TCGA training dataset. Univariate Cox regression indicated that high expression levels of HDAC1, HDAC3, HDAC7 and HDAC9 were associated with poor OS of glioma, and elevated expression levels of HDAC4, HDAC5, HDAC6 and HDAC11 were associated with a favorable prognosis of glioma (Figure 2A). HDAC2, HDAC8 and HDAC10 seemed not to be related to prognosis. Furthermore, LASSO regression identified six HDAC genes (HDAC1, HDAC3, HDAC4, HDAC5, HDAC7 and HDAC9) that entered the final model (Figure 2B and 2C). The risk score of each sample was calculated according to the following formula: risk score = 0.179 × HDAC1 expression + 0.502 × HDAC3 expression − 0.671 × HDAC4 expression − 0.567 × HDAC5 expression + 0.488 × HDAC7 expression + 0.216 × HDAC9 expression (Supplementary Table 3). The glioma patients were categorized into a high-risk group and a low-risk group using the median risk score (−0.360). The Kaplan–Meier curve showed that the high-risk group had a worse OS than the low-risk group (*P* < 0.001, Figure 2D). The risk score and survival time were separately distributed (Figure 2E). PCA showed two obvious risk distribution patterns (Figure 2F).

Patients from the CGGA dataset were used to validate the calculated risk score, and they were also separated into high- and low-risk groups according to the median calculated using the formula established in the TCGA training set. Similarly, survival analysis suggested that the high-risk group had a worse OS than the low-risk group (*P* < 0.001, Figure 2G), and the risk score and survival time were also visually scattered (Figure 2H). Likewise, PCA showed two-dimensional distribution patterns (Figure 2I). The AUCs of 1 year, 2 years and 3 years were 0.873, 0.884 and 0.904 in the TCGA training set, respectively (Figure 2J). The AUCs of 1 year, 2 years and 3 years were 0.705, 0.765, and 0.761, respectively, in the CGGA validation set (Figure 2K).

### Stratified analysis

**Figure 3:**
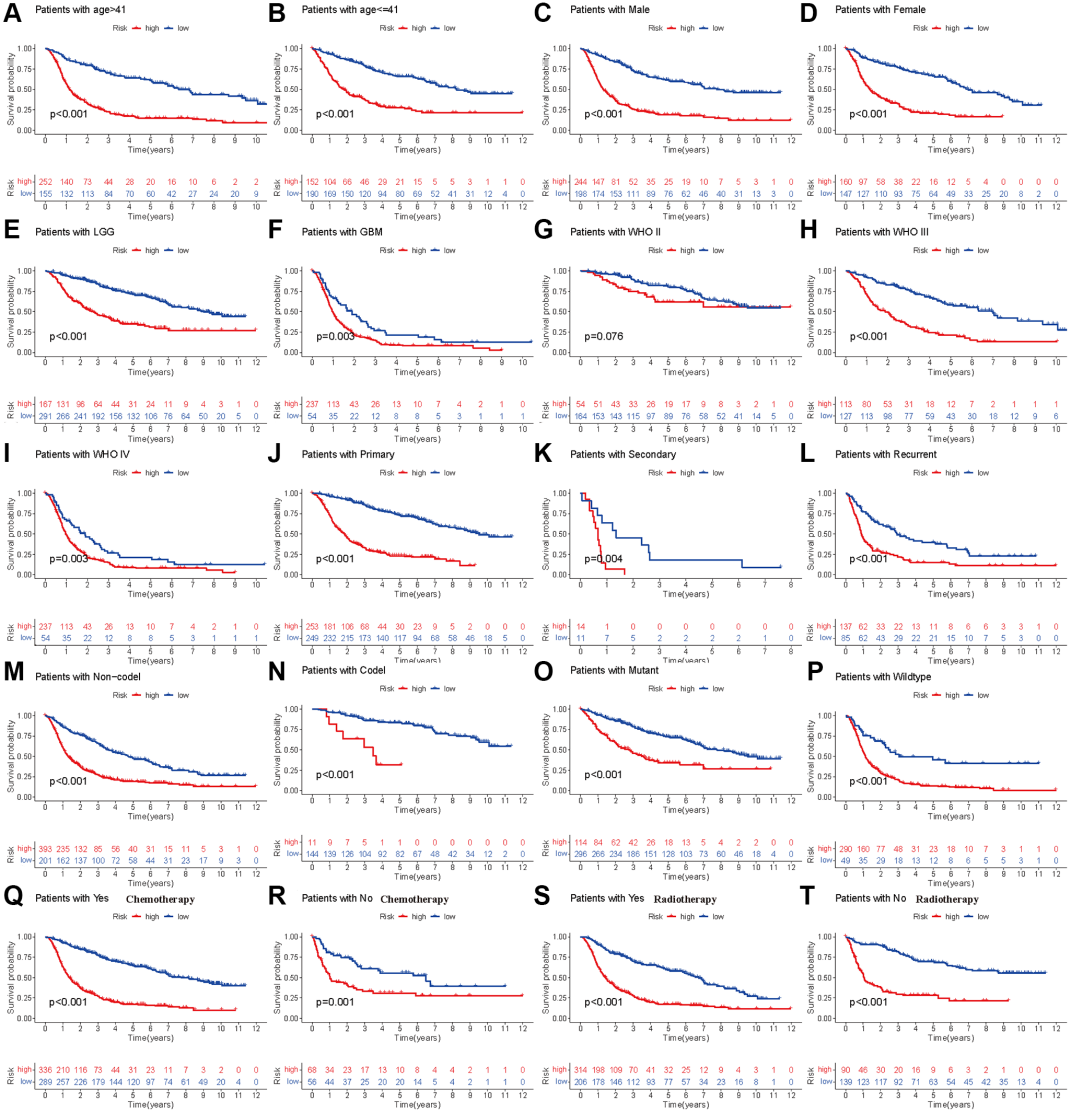
**Stratified analyses of established HDAC-related genes prognostic model in TCGA.** (**A** and **B**) Age (>41 vs. ≤41). (**C** and **D**) Gender (male vs. female). (**E** and **F**) Histology (LGG vs. GBM). (**G**–**I**) WHO stage (II, III and IV). (**J**–**L**) Type of tumors (Primary, secondary vs. recurrent). (**M** and **N**) 1p19q (Non-codel and codel). (**O** and **P**) mutant and wildtype. (**Q** and **R**) Radiotherapy (Yes vs. No). (**S** and **T**) Chemotherapy (Yes vs. No).

To further validate the prognostic model in different subgroups of glioma patients, we performed stratified analysis in different subpopulations. We found that low-risk patients based on the risk score had prolonged OS compared with the high-risk group, which was not affected by age, sex, histology, occurrence type, IDH codeletion status, 1p19q mutation, previous history of radiotherapy or chemotherapy (Figure 3A–3F, Figure 3H–3T). However, the OS showed insignificant differences for glioma patients with WHO grade II (Figure 3G), which means that the developed risk score may be inappropriate in such a subpopulation.

### Clinical correlation and independent analysis

**Figure 4:**
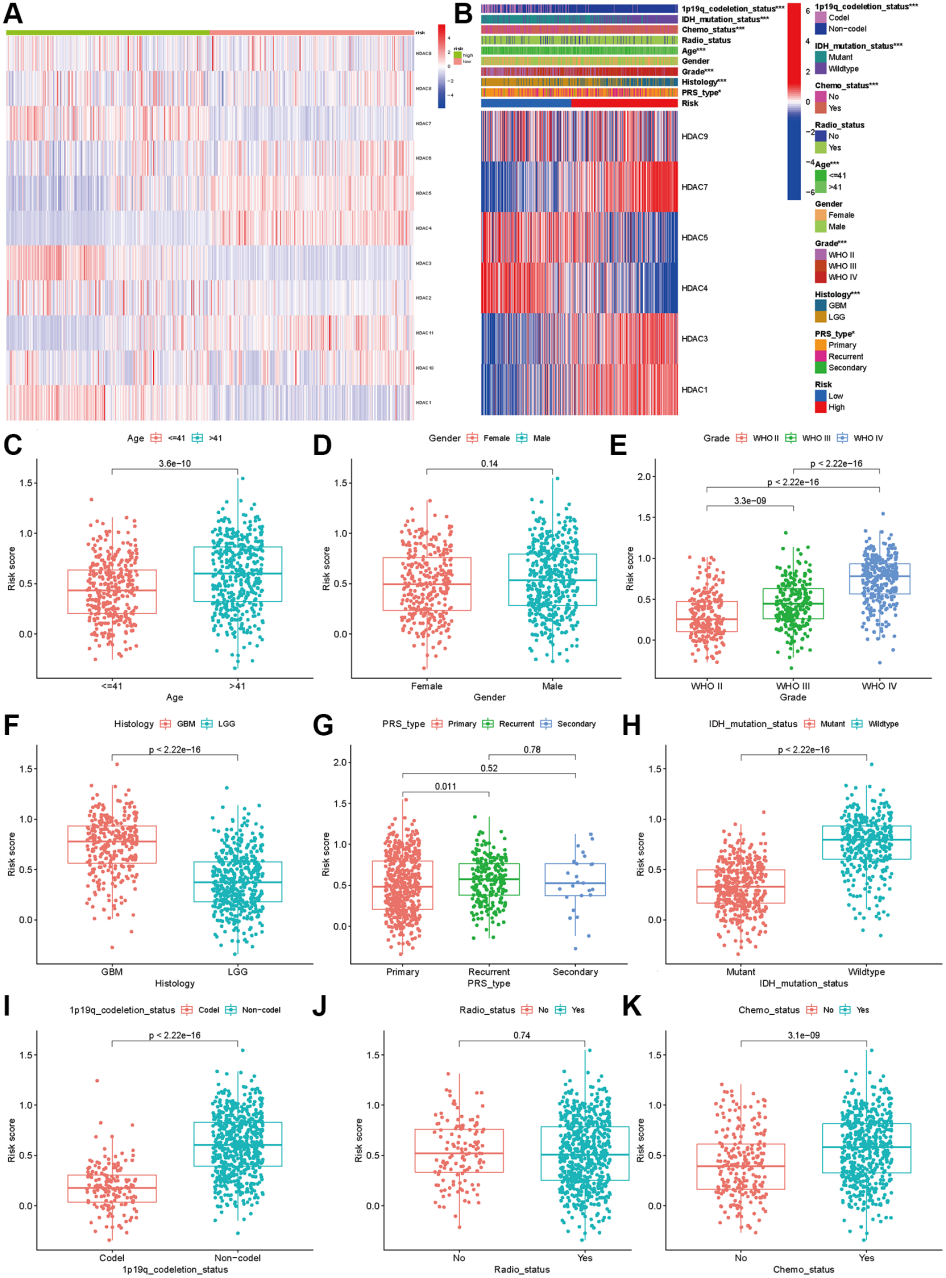
**Association between HDAC genes and clinical characteristics in glioma patients.** (**A**) Heatmap indicated the expression of HDAC genes between two subclasses. (**B**) Heatmap of associations among risk stratifications and clinical parameters and six HDAC genes expression. Comparisons of risk score among different clinical parameters: (**C**) age (>41 vs. ≤41), (**D**) gender (male vs. female), (**E**) WHO stage (II, III, IV). (**F**) histology (LGG vs. GBM). (**G**) PRS type (primary, recurrent, and secondary). (**H**) IDH mutation status (mutant vs. wild type). (**I**) 1p19q codeletion status (codel vs. non-codel). (**J**) radiotherapy status (No vs. Yes). (**K**) chemotherapy (No vs. Yes).

The analysis of expression differences indicated that all eleven HDAC genes showed significant differences between the two subclasses (Figure 4A). The chi-square test indicated that the high-risk patients tended to be GBM, WHO III/IV, older, re-occurrence or secondary, and have IDH mutation and 1p19q codeletion (*P* < 0.05). No significant differences between the high- and low-risk groups were observed for the radiotherapy ratio and sex ratio (*P* > 0.05, Figure 4B).

We compared the expression levels of the six HDAC genes included in the prognostic model and found that HDAC1 (*P* < 0.001), HDAC3 (*P* < 0.001), HDAC7 (*P* < 0.001) and HDAC9 were highly expressed in the high-risk group, while HDAC4 (*P* < 0.001) and HDAC5 (*P* < 0.001) were expressed at low levels in the high-risk group (*P* < 0.001). Then, we compared the risk score differences among the different clinical parameters. Our results indicated that patients >41 years old, advanced WHO stage and higher grade had higher risk scores (*P* < 0.005, Figure 4C–4F). Patients with IDH mutation and 1p19q codeletion had lower risk scores (*P* < 0.05, Figure 4H and 4I). However, the risk score showed no significant differences among different sexes (Figure 4D), recurrent or secondary (Figure 4G), or radiotherapy status (Figure 4J). Patients who received chemotherapy also had higher risk scores than those without chemotherapy (Figure 4K).

**Figure 5:**
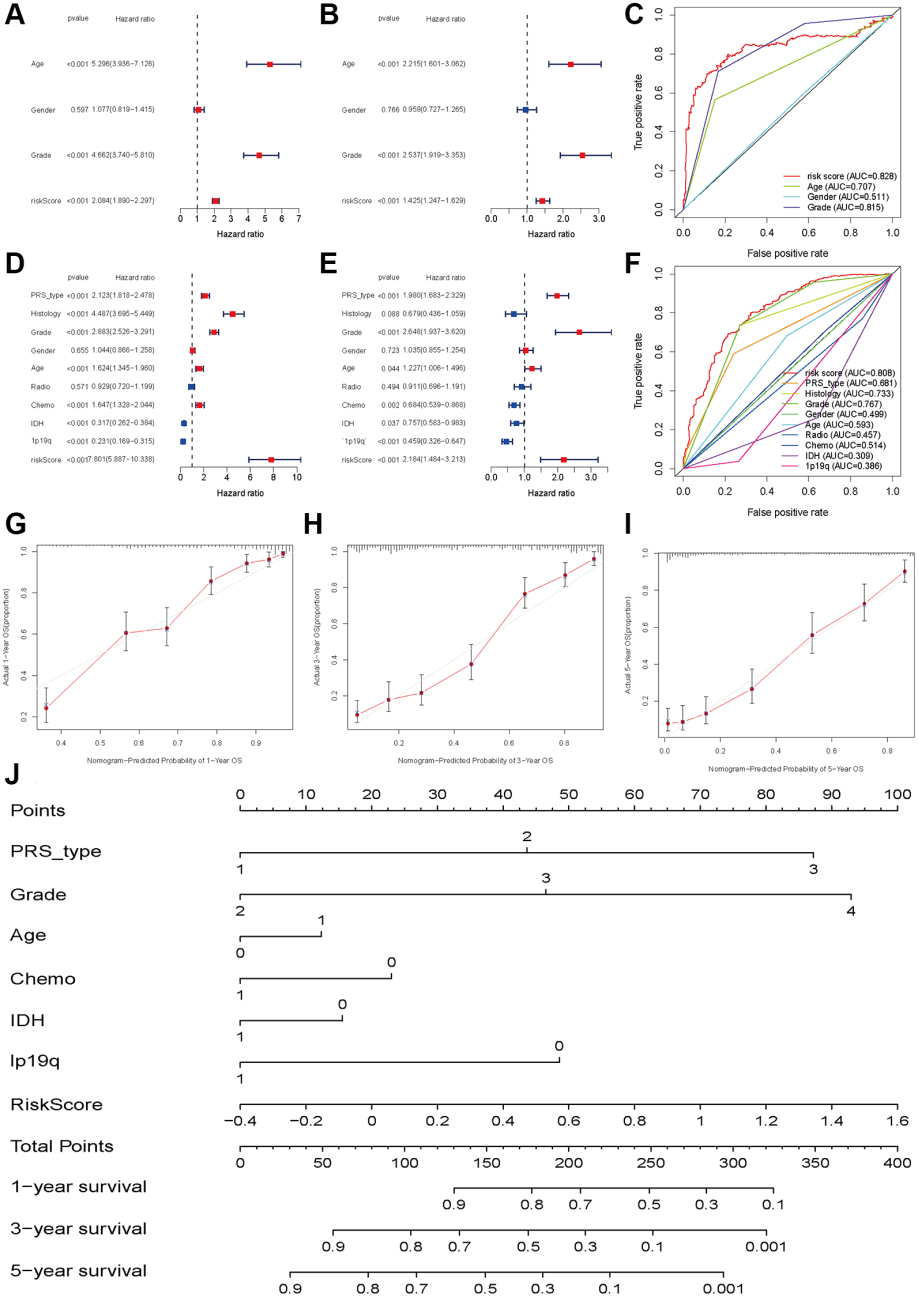
**Independent prognosis analyses of HDAC-related genes model.** (**A** and **B**) univariate and multivariate cox regression of risk score based on HDAC genes in TCGA. (**C**) The receiver operating characteristic curve of risk score for predicting 5-year survival rate in TCGA. (**D** and **E**) univariate and multivariate cox regression of risk score based on HDAC genes in CGGA. (**F**) The receiver operating characteristic curve of risk score for predicting 5-year survival rate in CGGA. (**G**–**I**) Calibration curves of 1-eyar, 3-year, and 5-year OS in TCGA. (**J**) Nomograph model established in CGGA cohort.

To investigate whether the risk score was an independent prognostic factor for glioma patients, we performed univariate and multivariate Cox regression in the TCGA training set and CGGA validation set. In the TCGA dataset, univariate and multivariate Cox regression analyses indicated that the risk score was associated with OS in glioma patients (univariate: HR = 2.084, 95% CI: 1.890–2.297, *P* < 0.001, Figure 5A; multiple: HR = 1.425, 95% CI: 1.247–1.629, *P* < 0.001, Figure 5B). The ROC results showed that the risk score had optimal predictive ability (AUC = 0 .828) for 5-year OS (Figure 5C). Similarly, an elevated risk score was also associated with a poor OS (univariate: HR = 7.801 95% CI: 5.887–10.338, *P* < 0.001, Figure 5D; multiple: HR = 2.184, 95% CI: 1.484–3.213, *P* < 0.001, Figure 5E). The AUC was 0.808, which was higher than that of any of the other clinical parameters (Figure 5F). In addition, recurrence, advanced grade and age were also risk factors for poor OS, while patients who received chemotherapy and had IDH mutations and 1p19q codeletion had a favorable OS (Figure 5E). The calibration plots were presented in Figure 5G–5I. The Nomograph was shown in Figure 5J.

### Functional and pathway enrichment and mutation analyses

**Figure 6:**
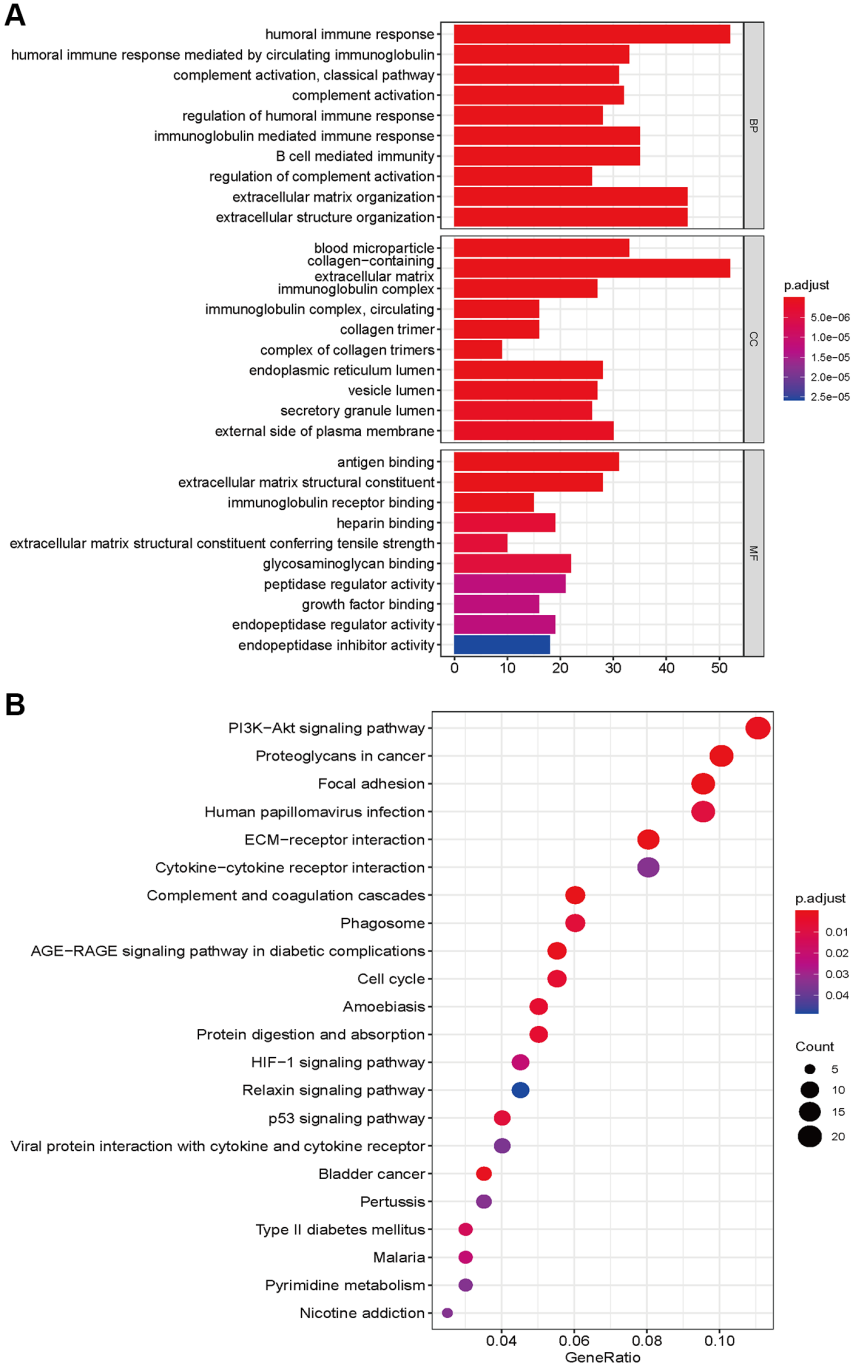
GO enrichment (**A**) and KEGG pathways analysis (**B**) based on differently expressed genes between high- and low risk groups.

To explore the functional and pathway enrichment of the high- and low-risk groups, we performed GO and KEGG analyses. We first identified genes differentially expressed between the high- and low-risk groups (log fold change >1, *P* < 0.05). We finally identified 2598 differentially expressed genes, including 1723 upregulated genes and 875 downregulated genes, in the high-risk groups (Supplementary Table 4). GO enrichment analysis indicated that the high-risk group was mainly enriched in immune-related functions in biological processes, collagen and lumen in cellular components, antigen binding, extracellular matrix structural constituents, regulators, receptors and inhibitor binding in molecular functions (Figure 6A). The KEGG pathway analysis showed that the high-risk group was involved in the PI3K-Akt signaling pathways, AGE-RAGE signaling, HIF-1 signaling, relaxin signaling, and p53 signaling. Focal adhesion, ECM-receptor and cytokine–cytokine receptor interactions, the cell cycle and pyrimidine metabolism were also significantly enriched (Figure 6B). The occurrence of glioma requires the integration of multiple molecular functions and signaling pathways.

**Figure 7:**
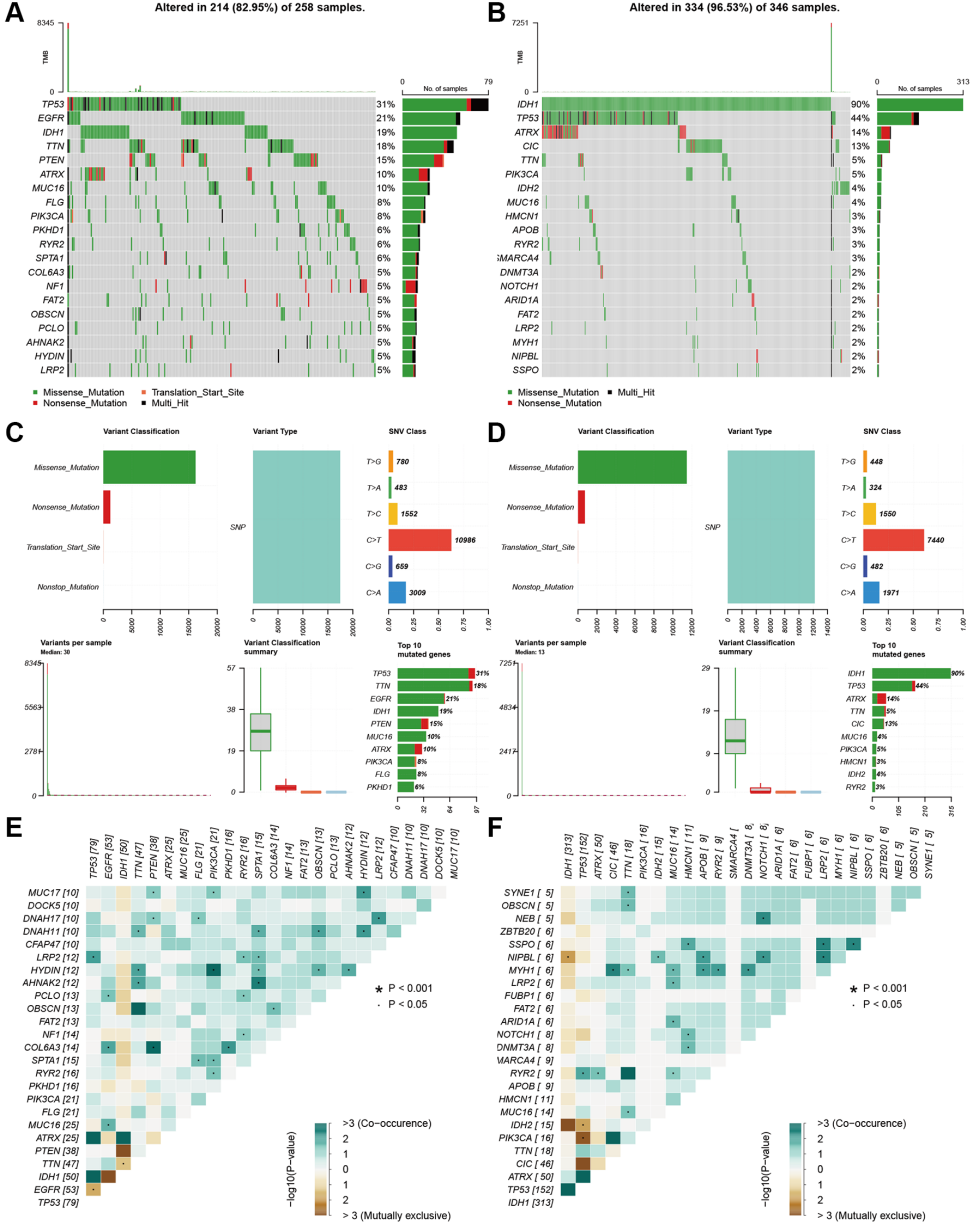
**Landscape of mutation profiles between high- and low-risk groups.** (**A** and **B**) Waterfall plots of mutation information in each sample. (**C** and **D**) Bar graph of variant classification. (**E** and **F**) somatic interactions plot (co-occurrence and exclusive).

We also explored the gene mutation differences between the high-risk and low-risk groups. Our results indicated that there were significant differences in gene mutations between the high- and low-risk groups. The high-risk group showed high gene alterations in EGFR, PTEN, FLG, and PKHD1 (Figure 7A), while the gene alteration rates of IDH, ATRX, and CIC were higher in the low-risk group than in the high-risk group (Figure 7B). The high- and low-risk groups showed similar results in variant classification, variant type, and SNV class (Figure 7C and 7D). Furthermore, HYDIN-PI3CA, AHNAK2-SPTA1, COL6A3-PTEN, and IDH1-TP53 showed high co-occurrence, and PTEN-TTN and IDH1-EGFR were mutually exclusive in the high-risk groups. SSPO-HMCN1, LRP2, NIPBL, MYH1-CIC, TTN, MUC16, APOB, RYR2, DNMT3A, NIPBL-IDH2, APOB, NOTCH1, and LRP2 showed high co-occurrence (Figure 7E). IDH2-TP53, PI3CA-TP53, and CIC-TP53 were mutually exclusive in the low-risk group (Figure 7F).

### Immune filtration, tumor microenvironment, and drug sensitivity analysis

**Figure 8:**
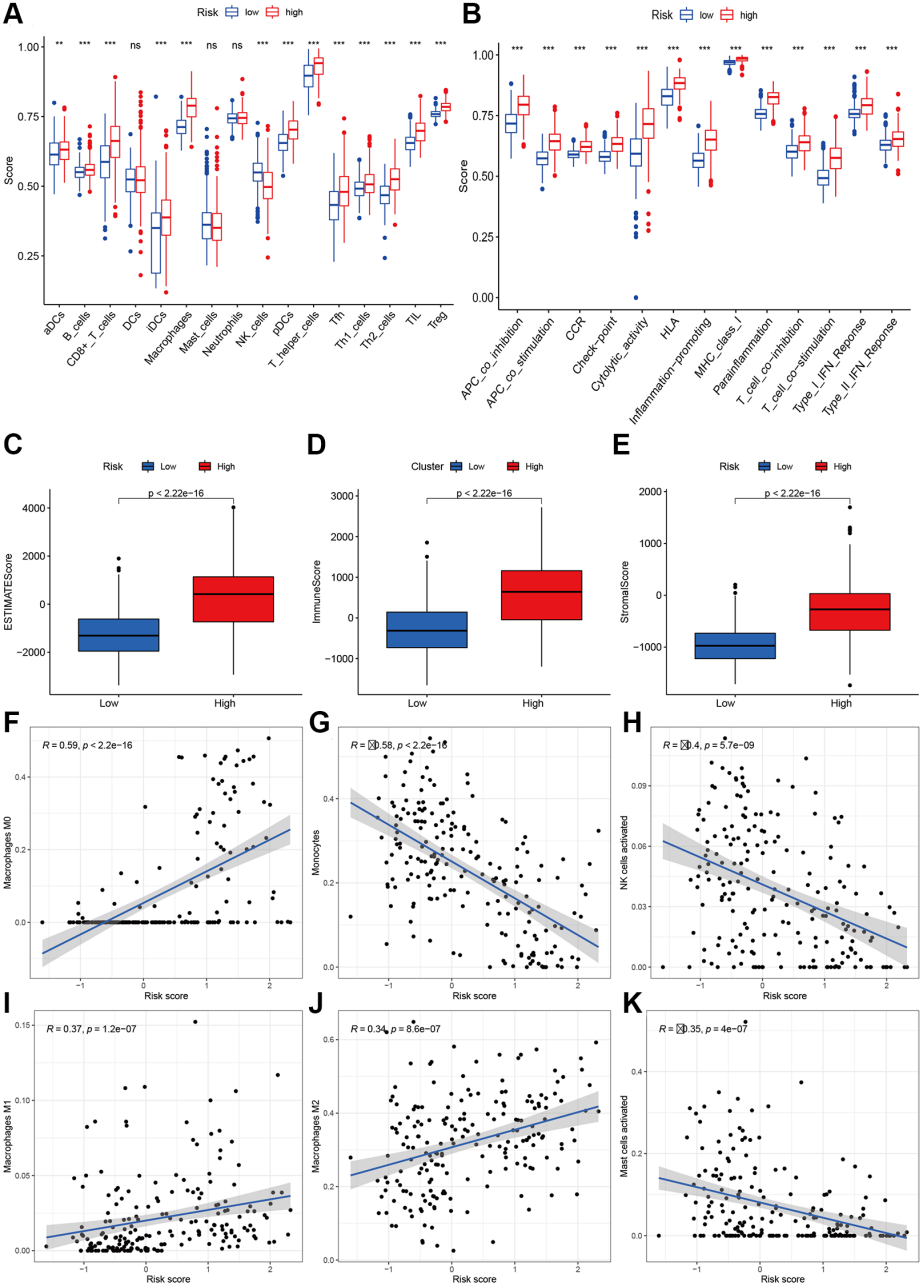
**Immune status analysis between high- and low-risk group.** (**A**) The ssGSEA scores of immune cells. (**B**) The ssGSEA scores of immune-related functions. (**C**–**E**) Comparisons of Estimated, immune and stromal score between high-and low-risk group. (**F**–**K**) Correlation between risk score and immune markers (Macrophages M0, Monocytes, NK cells activated, Macrophages M1, M2, and Mast cells activated) in glioma patients.

**Figure 9:**
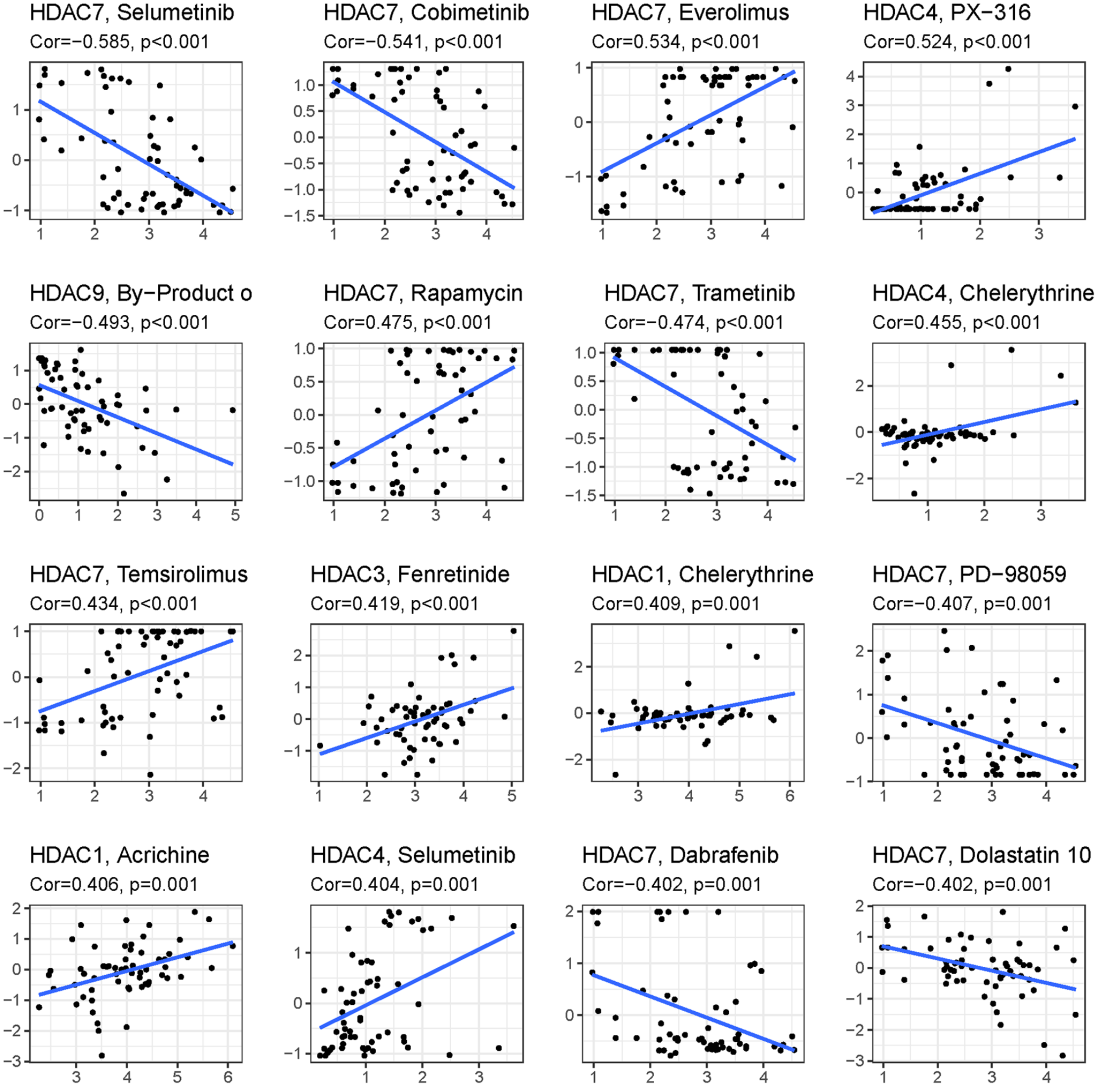
Top 16 kinds of drug associated with HDAC member.

To explore the immune response difference between the high- and low-risk groups, we compared the immune filtration cells and immune-related pathways between the high- and low-risk groups. Our results indicated that aDCs, B cells, CD8+ T cells, iDCs, macrophages, NK cells, pDCs, T helper cells, Tfh cells, Th1 cells, Th2 cells, TILs, and Tregs had higher proportions in the high-risk group, while no significant differences were observed for DCs, mast cells or neutrophils (Figure 8A). All immune-related pathways were highly enriched in the high-risk group (Figure 8B). We also found that the ESTIMATE score, immune score, and stromal score were higher in the high-risk group than in the low-risk group (Figure 8C–8E). Pearson correlation analysis indicated that M0, M1, and M2 macrophages showed positive associations with the risk score (Figure 8F, 8I, and 8J), while monocytes, activated NK cells, and activated mast cells showed negative associations with the risk score (Figure 8G–8K). We also identified some small molecule compounds that may guide chemotherapy for glioma (Figure 9).

### Validation of HDAC genes in glioma and nontumor tissue

**Figure 10:**
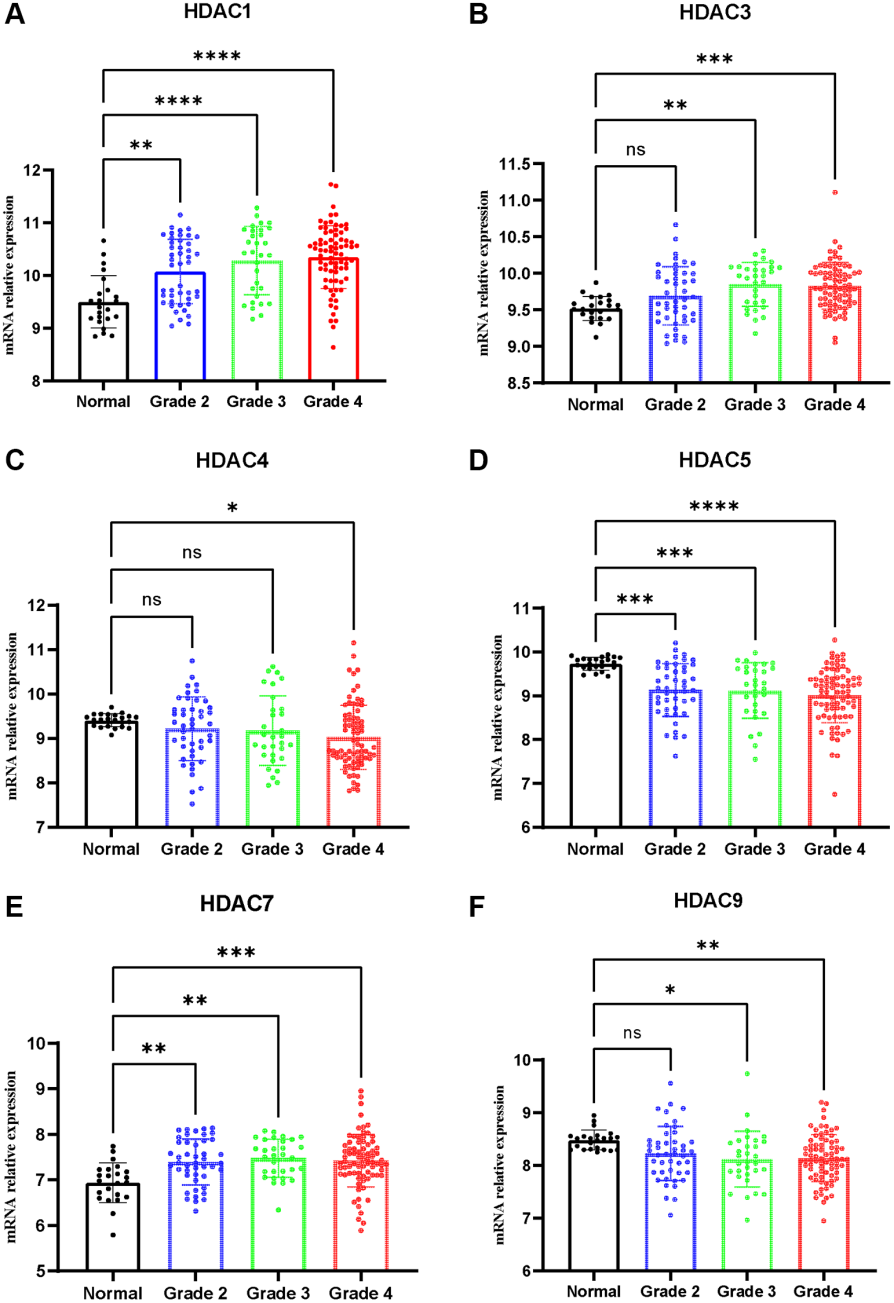
**Expression of HDAC genes in glioma and non-tumor tissue.** (**A**) HDAC1, (**B**) HDAC3, (**C**) HDAC4, (**D**) HDAC5, (**E**) HDAC7, (**F**) HDAC9.

qPCR was adopted to detect the expression of HDAC1, HDAC3, HDAC4, HDAC5, HDAC7 and HDCA9 in nontumor and glioma tissues. The results are presented in Figure 10. The expression levels of HDAC1 (Figure 10A), HDAC3 (Figure 10B), HDAC7 (Figure 10E), and HDAC9 (Figure 10F) were significantly elevated in the glioma patients compared with the nontumor group. However, the expression levels of HDAC4 and HDAC5 were lower in the glioma patients than in the nontumor control groups (Figure 10C and 10D). This result is consistent with the role of these HDAC genes in glioma prognosis.

## DISCUSSION

HDACs, as key enzymes that catalyze the acetylation of histones, are involved in many processes, such as the growth and proliferation of malignant tumor cells and gene expression regulation [16]. Epigenetic research on tumor occurrence and development has gradually attracted wide academic attention worldwide. Most of the current research focuses on the chemical and structural modification of the existing antitumor drugs with HDAC inhibitory activity to enhance the therapeutic effect of the drugs and alleviate their toxicity and side effects [17]. There are currently few antitumor drugs designed to act on specific targets and specific pathways. At the same time, it is essential to explore the molecular signatures for a better understanding of the biological relationship between tumor genotype and phenotypes.

In the present study, we found that glioma patients can be divided into two subclasses based on the expression patterns of eleven HDAC genes, and patients from the two subclasses had markedly different survival outcomes. Then, using six HDAC genes (HDAC1, HDAC3, HDAC4, HDAC5, HDAC7, and HDAC9), we established a prognostic model for glioma patients, and this prognostic model was validated in an independent cohort population. Furthermore, the calculated risk score from the expression of the six HDACA genes was found to be an independent prognostic factor, able to accurately predict the five-year overall survival of glioma patients. High-risk patients can be attributed to changes in multiple complex functions and molecular signaling pathways, and the gene alterations between high- and low-risk patients were significantly different. We also found that different survival outcomes of high- and low-risk patients could be involved in the differences in immune filtration levels and the tumor microenvironment. Subsequently, we identified several small molecular compounds that could be favorable for glioma patient treatment. Finally, we validated the expression levels of HDAC genes from the prognostic model using glioma and nontumor tissue samples. Our study provides new and simple molecular subtypes and prognostic prediction methods and adds to our understanding of the biology and molecular mechanisms of glioma.

We included six HDAC genes in the established prognostic model. HDAC1 and HDAC3 are Class I HDACs. Previous studies have indicated that HDAC1 is overexpressed in diverse human malignancies, such as prostate cancer, breast cancer, liver cancer, and lung cancer [18–21]. HDAC1 is also highly expressed in glioma tissue, and high expression of glioma is associated with glioma cell proliferation, migration, invasion, angiogenesis, and a poor prognosis [22]. In addition, has been suggested that increased activation of HDAC1/2/6 and Sp1 underlies therapeutic resistance and tumor growth in glioblastoma [23]. We also found that the expression of HDAC1 was elevated in glioma tissues and was associated with a poor prognosis.

HDAC3 has become a focus of recent research, and many scholars worldwide have found that it plays a role in carcinogenesis in human tumors. After the expression of HDAC3 is reduced by inhibitors, the growth and invasive abilities of human glioma cells are significantly weakened, which provides a new target for cancer treatment [24].

HDAC4, HDAC5, HDAC7, and HDAC9 are Class II HDACs [25]. HDAC4 is frequently dysregulated in human malignancies, and we also confirmed its downregulated expression in glioma tissues. However, previous studies reported that HDAC4 was significantly upregulated in glioma tissues. The proliferation, adenosine triphosphate (ATP) levels and invasion ability were substantially enhanced in U251 cells with HDAC4 overexpression and suppressed in U251 cells with HDAC4 knockdown compared with U251 cells transfected with a negative control [26, 27]. This may be associated with glioma grade, stage and histology, and further research is needed. Similar to HDAC4, HDAC5 was also found to be expressed at low levels in glioma tissue.

HDAC7 plays an oncogene role in glioma. It was reported that ZNF326 could activate HDAC7 transcription by binding to a specific promoter region via its transcriptional activation domain and zinc-finger structures in glioma cells [28]. Furthermore, ZNF326 was not only highly expressed in glioma but was also positively correlated with the expression of HDAC7, which identified the oncogenic role of HDAC7 [29].

HDAC9, like most class II HDACs, has a conserved histone deacetylase domain, catalyzes the removal of acetyl moieties from the N-terminal tail of histones, and possesses a long regulatory N-terminal domain that interacts with tissue-specific transcription factors and corepressors. The amino-terminal domain contains highly conserved serine residues that are subjected to phosphorylation [30]. Signal-dependent phosphorylation of HDAC9 is a critical event that determines whether it is localized in the cytoplasm or nucleus. High HDAC9 expression has been reported in many cancers [31–33]. In glioma, the high expression of HDAC9 can promote proliferation and tumor formation and accelerate the cell cycle in part by potentiating EGFR signaling pathways [34].

With the emerging and rapid development of disciplines such as structural biomechanics and computer-aided drug design, the development of new HDAC inhibitors with antitumor activity targeting HDACs is bound to have a very broad application space and developmental prospects.

We noticed that a recent study also evaluated the role of HDCA genes in glioma [35]. There are several marked differences between our study and their study. (1) We used HDAC genes for clustering glioma and found that two clusters were obtained. Then, we developed and validated a prognostic model using the CGGA and TCGA datasets, evaluated the correlations among the risk score, immune infiltration and clinical characteristics, and established a tool for evaluating the prognosis of individuals. We also evaluated the gene alterations and different functional and pathway enrichment between the two risk groups. Finally, we validated the expression of the HDAC genes in the GEO dataset. These analyses were not performed in Li’s study in 2022. Li’s study evaluated the expression differences of HDAC genes between tumor and normal tissues and the correlation of each individual HDAC gene with the prognosis, quite different from our study.

The present study has several limitations. One is that the established model needs to be validated in other cohorts. The other limitation is that we did not explore the specific molecular mechanism of this model in glioma, and some results need to be verified *in vitro* and *in vivo*.

In summary, our results reveal the clinical utility and potential molecular mechanisms of HDAC genes in glioma. A model based on six HDAC genes (HDAC1, HDAC3, HDAC4, HDAC5, HDAC7, and HDAC9) can predict the overall survival of glioma patients well and these genes are potential therapeutic targets. Future research should validate this model in a large cohort, and experiments *in vivo* and *in vitro* will improve our understanding of the molecular mechanisms of glioma.

## Supplementary Materials

Supplementary Table 1

Supplementary Table 2

Supplementary Table 3

Supplementary Table 4
