# Supplementary material for: Comprehensive analysis of histone deacetylases genes in the prognosis and immune infiltration of glioma patients
Source: Aging (Albany NY). 2022 May 11;14(9):4050–68. doi: 10.18632/aging.204071 (PMC9134955; doi:10.18632/aging.204071)
Supplement: Supplementary Table 3 [file aging-14-204071-s003.pdf]

SUPPLEMENTARY TABLE

Supplementary Table 3. Coefficient of HDAC in the included model.

Gene	Coefficient	HR
HDAC9	0.215663405	1.240685
HDAC7	0.487987014	1.629034
HDAC5	−0.567053902	0.567194
HDAC4	−0.670958585	0.511218
HDAC3	0.502378317	1.652647
HDAC1	0.179033415	1.196061
